# First demonstration of machine-designed ultra-flat, low-cost directive antenna

**DOI:** 10.1038/s41598-020-67354-2

**Published:** 2020-06-29

**Authors:** Marcello Zucchi, Giorgio Giordanengo, Marco Righero, Giuseppe Vecchi

**Affiliations:** 1 Antenna and Electromagnetic Compatibility Laboratory, Department of Electronics and Telecommunications, Politecnico di Torino, 10124 Turin, Italy; 2 Antenna and Electromagnetic Compatibility Laboratory, Advanced Computing and Applications, Fondazione LINKS, 10138 Turin, Italy

**Keywords:** Electrical and electronic engineering, Design, synthesis and processing

## Abstract

In this paper, we present a fully automated procedure for the direct design of a novel class of single-feed flat antennas with patterning of a conductive surface. We introduce a convenient surface discretization, based on hexagonal cells, and define an appropriate objective function, including both gain and input matching requirements. The reference geometry is constituted by a very thin, single feed-point square panel. It features a backing metal plate (“ground”) and a top conductive layer, which is automatically patterned to achieve the desired radiation and input matching properties. The process employs an evolutionary algorithm combined with a boundary element electromagnetic solver. By applying this method, we designed an antenna tailored to the 2.4 GHz ISM frequency band, with a size of $24\,\hbox {cm} \times 24\,\hbox {cm}$, i.e., $2 \times 2$ wavelengths and an height of 4 mm, or 0.03 wavelengths. Measured data confirmed the expected high gain (13 dBi), with a remarkable aperture efficiency (higher than 50%, including losses), thus validating the proposed approach.

## Introduction

The design of flat directive antennas is characterized by the challenge of combining high gain, small losses, as well as a reduced volume occupation. Over the years, this prompted the development of different methods and technologies, notably arrays and leaky-wave antennas.

The last decade has seen the appearance of flat antennas based on metasurface paradigms^1,2^. In this class of radiating structures, radiation is obtained by the excitation of a large number of sub-wavelength passive elements, called “atoms”. These “atoms” are commonly realized by means of shaped metal patches printed on a dielectric (grounded) slab. This allows for inexpensive PCB-technology fabrication over a wide range of frequencies. Furthermore, these antennas do not require a beam-forming network, therefore reducing ohmic losses. At present, metasurface antennas represent the most viable low-loss, low-cost, light-weight alternative to the established array-based design. For these reasons, the design of single-feed-point metasurface antennas has been investigated both theoretically^3–5^ and experimentally^6–8^.

In this work we address the design of flat directive antennas based on a PCB-realizable structure. We propose and adopt the radical concept of *direct* design, without any assumption on, or approximation of the radiation mechanism. The proposed automated procedure is based on evolutionary fitting to reach a specified goal, combined with full-wave solution of the associated electromagnetic problem.

**Figure 1:**
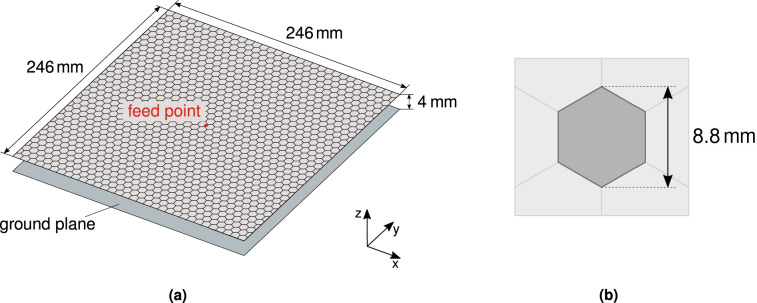
Antenna architecture: (**a**) full structure: the pictured top layer shows the hexagonal tiling; (**b**) hexagonal pixels (*hexapix*) of patterning.

The antenna structure consists of a very thin, single-feed panel. It comprises a backing metal plate (the ground plane) and an overlying patterned conductive layer (Fig. 1).

A key ingredient is the novel hexagonal-pixel (*hexapix*) tiling structure used to conveniently discretize the radiating surface. The patterning is obtained by binary inclusion/removal of *hexapix*, allowing the structure to evolve to its optimal shape. All possible configurations are included, down to the spatial granularity defined by the hexapix size. The binary-based evolutionary design is naturally achieved by a Genetic Algorithm (GA). The discrete nature of the patterning allows efficient repeated simulations via boundary-element method, as outlined in the literature^9–13^. The employed boundary-element simulation approach is standard and is usually called Method of Moments (MoM) in the antenna community^14^. The optimization relies on an appropriate definition of the objective function. The proposed objective function has a clear physical meaning, and allows to simultaneously take into account both gain and input matching requirements.

In the past, similar approaches—based on square cells—have been applied to the optimization of compact (i.e., electrically small) microstrip antennas^9,12,15–17^, and to unit-cell design for Frequency Selective Surfaces^18^ and optical metasurfaces^19^. To the best of the authors’ knowledge, no previous work has achieved, or even considered, the direct design of directive antennas (with gain above 10 dBi), with dimensions well beyond resonant size.

The proposed method is then applied to the design of a broadside antenna working in the 2.4 GHz ISM frequency band, with a size of $24\,{\mathrm{cm}} \times \,24\,{\mathrm{cm}}$ ($2 \lambda \times 2 \lambda$ at the working frequency) and with a thickness of 4 mm.

Measurements performed on the fabricated prototype confirmed the simulated performance, with high gain and remarkable efficiency both with regard to aperture illumination and losses.

To the best of the authors’ knowledge, this is the first time that the automated design of flat antennas of this size is reported in the open literature, and demonstrated experimentally. This has been possible by the innovation reported here, which can be summarized in the following items: (1) the overall architecture of the antenna, that is able to capture the relevant wave phenomena; (2) the *hexapix* paradigm, that leads to a directly realizable structure; (3) the theoretical work that leads to the formulation of an effective objective function for the machine design.

## Results

### Structure

The design of the antenna starts with the choice of its overall *architecture*. This step is crucial in obtaining design robustness, and must allow for direct fabrication with readily available PCB technology.

**Figure 2:**
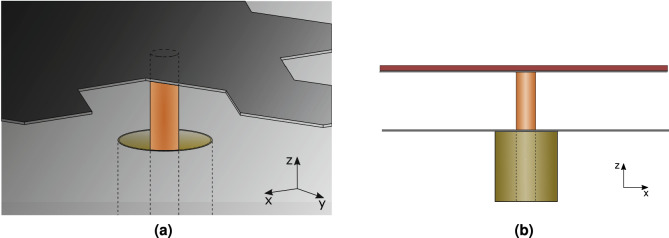
Detail of the coaxial feed: (**a**) perspective view; (**b**) side view.

The architecture in Fig. 1a was chosen; it fulfills the requirements for low-profile and low-cost. The machine design then consists in finding the pattern of the conductive layer. The latter is printed on a thin dielectric support, suspended above a metallic ground plane. The antenna is fed at the center by a coaxial connector through the ground plane, and the conductive layer is connected to the center conductor, as in Fig. 2. The overall structure is very thin, with a height $h\ll \lambda$, where $\lambda$ is the operational wavelength. All relevant sizes are reported in Fig. 1a.

The top conductive layer was partitioned into 1230 *hexapix* cells (Fig. 1b), resulting in a deeply sub-wavelength resolution for the patterning, $d \approx \lambda /28$. The advantage of the *hexapix* over the usual square cell consists in its ability to prevent isolated corner contacts, which represent a major cause of uncertainty during the manufacturing phase^20^. Indeed, in a previous work^21^ we used the square cells in the design of a similar antenna, with the same specifications and size. There, we found that it was impossible to satisfy both gain and input matching requirements, as a result of the excessive manufacturing sensitivity caused by metallic corner contacts.

### Design algorithm

We observe that in our approach each possible structure is described by a *binary string*. Therefore, it is natural to address the evolutionary design process via a Genetic Algorithm (GA). GA is a well known heuristic stochastic global optimization method based on principles derived from natural evolution. It targets non-convex and non-linear problems, such as those arising in practical electromagnetic design problems^22–24^.

Single elements of the search space are called *individuals* and consist of strings of parameters called *chromosomes* which encode all relevant features—in this case each representing a given patterning. These belong to sets called *populations*. The algorithm advances by coupling the elements within the population to produce the subsequent generation. This is done by means of operators inspired by natural processes: *selection* picks individuals from the population based on their fitness value, *crossover* creates a new individual by combining the genetic content of two parent individuals and *mutation* introduces variation in the genes randomly.

The design objective is expressed as the maximization of a *fitness* function, which gives the degree of goodness of a particular individual with respect to the set goals. The effectiveness of the objective (fitness) function is crucial in ensuring practical usefulness of any optimization result. To this end, our objective addressed both *gain*, and *input matching* of the antenna.

It appears natural to consider maximization of the gain along the desired direction of maximum radiation, here being the normal to the antenna face. However, we found that this choice was not optimal in the overall process. Analysis showed that this criterion was too strict in the early phases of the evolutionary design: individuals with high directivity but radiations somewhat off the maximum direction were excluded from further evolution, with detrimental effects. Therefore, we employed the more relaxed, gain-related *solid-beam efficiency*^25^. This quantity is defined as the relative fraction of power $\Delta P(\Delta \Omega )$ radiated in a given solid angle $\Delta \Omega$ around the desired beam direction. For our purpose, we used a normalized beam efficiency,

1 $$\begin{aligned} \hbox{BE}_{\Delta \Omega } = \frac{\Delta P(\Delta \Omega )}{P_{\mathrm{rad}}} \frac{4 \pi }{\Delta \Omega } \end{aligned}$$

where $P_{\mathrm{rad}}$ is the total radiated power. The solid angle $\Delta \Omega$ has the meaning of a “control volume”: when $\Delta \Omega$ tends to zero, the employed beam efficiency ${\mathrm{BE}}_{\Delta \Omega }$ recovers the common definition of directivity; $\Delta \Omega$ is one of the algorithmic design parameters, and it is apparent that it should be a fraction of the targeted beam-width (e.g. Half-Power Beam-Width, HPBW). The chosen value, after some experimentation, is reported in Table 1; the value indicated there assumes a conical region, $\Delta \Omega = \pi \,\Delta \theta ^2$, and $\Delta \theta$ is reported. Its comparison with the actual realized HPBW can be inferred from Table 2.

The second parameter is the *input matching* *T*. It is defined in the usual way as the ratio of the accepted power $P_{\mathrm{a}}$ to the incident power $P_{\mathrm{inc}}$,

2 $$\begin{aligned} T = \frac{P_{\mathrm{a}}}{P_{\mathrm{inc}}} = 1-|S_{11}|^2 \end{aligned}$$

where $|S_{11}|^2$ is the ratio between reflected and incident powers (with usual $50\,\Omega$ reference). Both indicators, ${\mathrm{BE}}_{\Delta \Omega }$ and *T*, are derived from full-wave simulation of the full structure for any concerned surface patterning.

It is important to note that the product of ${\mathrm{BE}}_{\Delta \Omega }$ and *T* has a direct physical interpretation, being proportional to the total *realized gain* of the antenna^25^. This suggests a practical way of combining the two objectives into a single fitness function. If considered in logarithmic (dB) format, one would have the sum of the two goal indicators, ${\mathrm{BE}}_{\Delta \Omega }$ and *T*. We thus defined the fitness function as a weighted product:

3 $$\begin{aligned} {\mathrm{fitness}}(T,{\mathrm{BE}}) = T^\alpha \cdot {\mathrm{BE}}_{\Delta \Omega }^\beta , \qquad \alpha + \beta = 2 \end{aligned}$$

This corresponds to a convex combination in logarithmic format, and allows more flexibility compared to the plain product. The weighting exponents $\alpha$ and $\beta =2-\alpha$ can be chosen so as to provide convergence toward a robust solution. Suitable values were found via extensive trials on the specific problem.

Finally, we remark that this objective function also possesses a physical upper bound. In fact, $T\le 1$, and

4 $$\begin{aligned} {\mathrm{BE}}_{\Delta \Omega } \le D_{\mathrm{max}}, \qquad D_{\mathrm{max}}= \dfrac{4\pi }{\lambda ^2}\,A_{\mathrm{geom}} \end{aligned}$$

where $D_{\mathrm{max}}$ is the maximum directivity achievable with the considered area. This corresponds to ideal unit ($100\%$) aperture efficiency (see below for definition).

In summary, the employed fitness function has a physical meaning, it includes *only two parameters* to be set ($\alpha$ and $\Delta \Omega$), and also possesses a physical upper bound. This is important to ensure a successful outcome.

With regard to the *stopping criterion*, it was decided to end the optimization when the best solution had not changed after a given number of generations $N_{\mathrm{stag}}$ (stagnation limit). In Table 1 the values of the main algorithmic parameters are reported.

**Table 1:** Algorithmic parameters and their values; GA parameters refer to common use^24^.

Parameter	Value
Population size	400
Chromosome length	615
Selection	Rank order
Crossover	Single point
Mutation rate	0.7%
Elite promotion	2
$N_{\mathrm{stag}}$	100
$\Delta \theta$	$5^\circ$
$\alpha$	1.4

### Simulation

The evaluation of the fitness function requires a full-wave electromagnetic analysis of the antenna. In principle, any simulation method and any implementation could be used. However, the binary nature of the patterning is especially suited to the use of a Boundary Element, Integral Equation approach; the related boundary value problem is called Electric Field Integral Equation (EFIE). For the numerical solution, the surface is discretized into triangular patches and the (equivalent) surface current is expressed as a linear combination of a set of local basis functions ${{\varvec{f}}}_n$ (Boundary Elements)^26^ defined on the triangular discretization,

5 $$\begin{aligned} {{\varvec{J}}}_{\mathrm{s}}({{\varvec{r}}}) \approx \sum _{n=1}^N I_n\;{{\varvec{f}}}_n({{\varvec{r}}}) \end{aligned}$$

This results in a linear system via Galerkin weighted-residual projection, historically called *Method of Moments* (MoM) in the antenna community,

6 $$\begin{aligned} {\mathbf{Z}} \,{\mathbf {I}}={\mathbf{V}} \end{aligned}$$

where ${\mathbf{Z}}$ is the system matrix, ${\mathbf{V}}$ is the vector of incident electric field and ${\mathbf {I}}$ is the vector of unknown coefficients $I_n$ to solve for. The EFIE-MoM results in a fully populated matrix ${\mathbf{Z}}$. However, as recognized by several works^10,12^, the binary operations involved in the geometrical description of the radiating surface allow a quick build of the system matrix at each iteration. This is obtained directly from the system matrix of the *mother* structure consisting of full metal.

The design goal included a linear polarization along the maximum radiation direction. This requirement was incorporated by simply enforcing structural symmetry, as the feeding is at the center of the structure. This was also effective in reducing the search space, as only half of the structure was considered.

### Results of design algorithm

**Figure 3:**
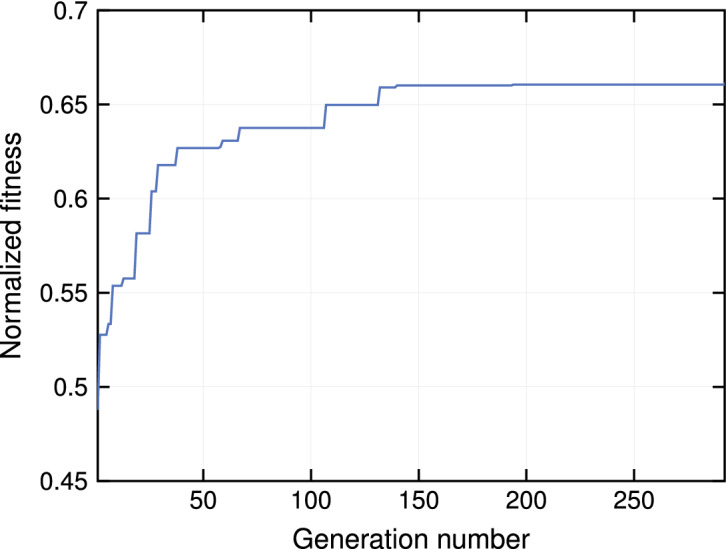
Convergence plot of the Genetic Algorithm. The plot reports the normalized fitness of the best individual for each generation. The fitness value is normalized to the physical upper bound, which corresponds to ideal impedance input matching and $100\%$ aperture efficiency, with the weighting exponents ($\alpha$, $\beta$) used in the design.

The evolution of the design algorithm can be observed in Fig. 3, in which the fitness value of the best individual is reported for each generation. The fitness value is normalized with respect to its physical upper bound. The algorithm took 292 generations to reach convergence (no improvement for 100 consecutive generations).

**Figure 4:**
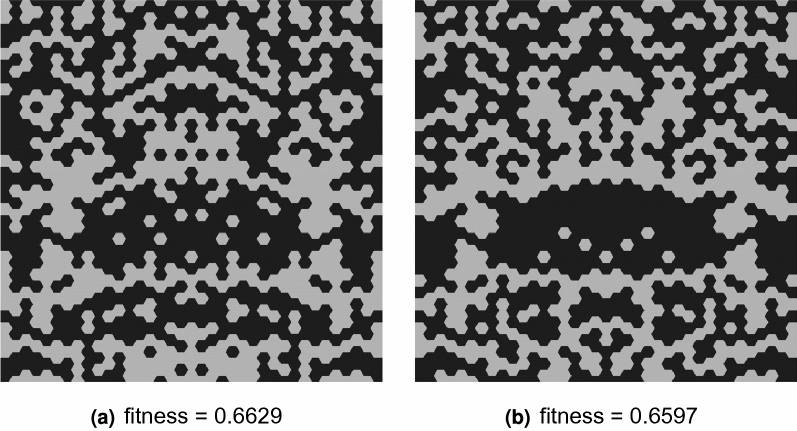
The (**a**) best and (**b**) second-best individuals after GA optimization, with their respective fitness values (normalized to the physical upper bound). Black areas represent metallic patterning. The best configuration (**a**) was chosen for fabrication and testing.

Once the design process ended, it provided a set of available structures. These correspond to the population of the last generation; each is characterized by a value of fitness. Fig. 4a shows the best and chosen structure. We found instructive to examine also the second-best solution, reported in Fig. 4b. It differs significantly in its geometric features, but performs comparably, as indicated by a fitness value close to the best one. This is intrinsic to the use of sub-wavelength details, and is also an indication of the robustness of the chosen architecture.

**Fig. 5:**
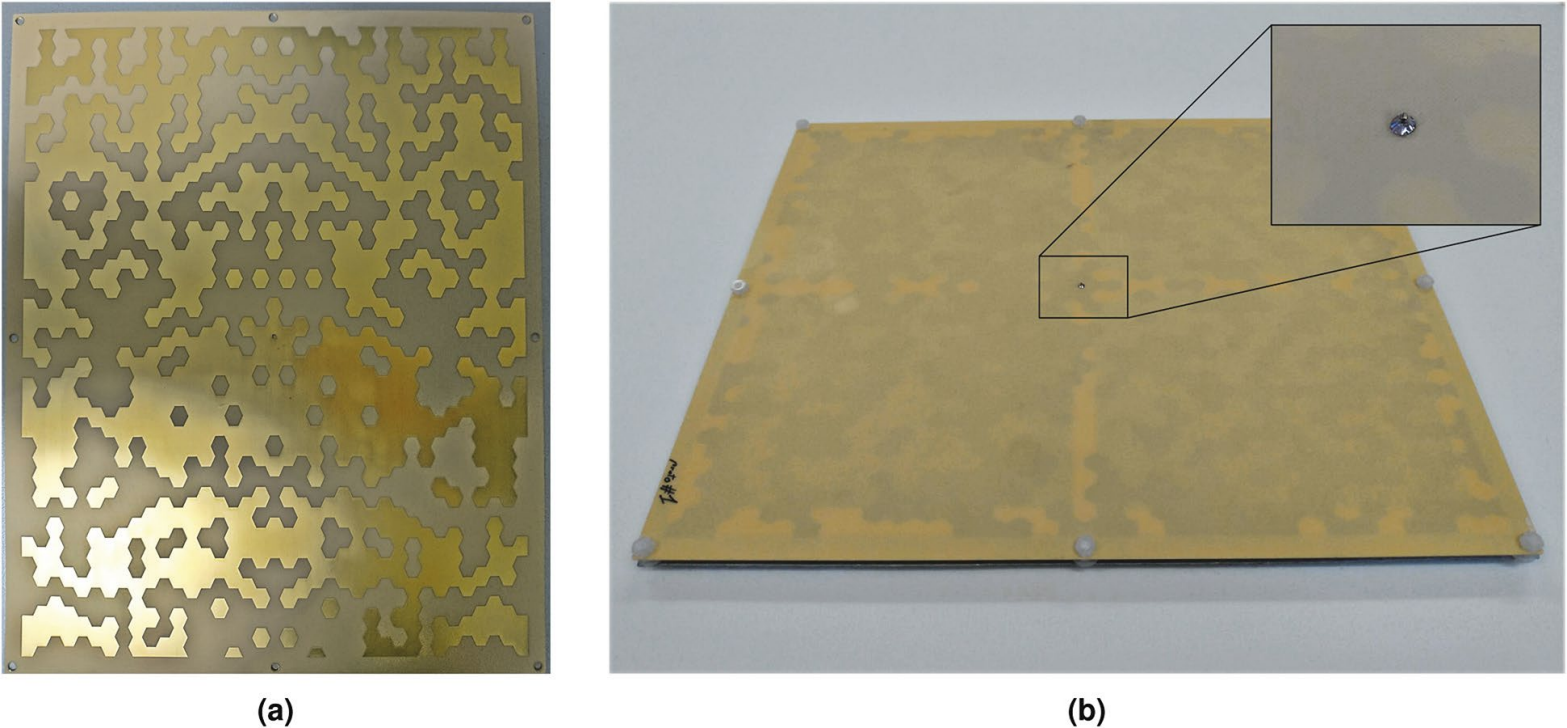
Fabricated antenna: (**a**) patterned top conductive layer, before assembly. The pattern is obtained by etching the copper on the dielectric support; (**b**) final assembly, obtained by mounting the top layer upside down above the ground plane (inset: detail of the central pin connection to the top layer).

The antenna was manufactured employing the best configuration, shown in Fig. 4a. It was fabricated using standard PCB technology. The upper, patterned conductive layer was etched on a dielectric I-Tera^27^ layer with a thickness of 0.254 mm (Fig. 5a). This dielectric-supported conductive layer was flipped back and then suspended above the metal ground plane by means of (eight) plastic spacers (Fig. 5b). The structure is fed by an SMA connector mounted on the back of the ground plane, with the center conductor connected to the patterned conductive layer. Due to the flip-back layout of the conductive layer, the connection to the coaxial pin was ensured via a plated through-hole and a small metal pad on the opposite face of the patterned layer. This choice allowed to carefully solder the pin to the latter pad, which is easily accessible (inset of Fig. 5b).

### Measured results

It is important to clarify that all measured results refer to actual parameters measured as per current standard^25^. In particular, we remark that the modified gain indicator (1) was used in the optimization process, but not in the measurements. Also, during the design process, the conductors were considered lossless, but the measured gain does of course account for the actual losses of the employed conductor materials.

In the evaluation of flat antennas performance, a relevant figure of merit is given by the *aperture efficiency* $\varepsilon _{\mathrm{ap}}$, which quantifies how much of a surface effectively radiates. It is defined as

7 $$\begin{aligned} \varepsilon _{\mathrm{ap}} = \frac{A_{\mathrm{eff}}}{A_{\mathrm{geom}}} \end{aligned}$$

where $A_{\mathrm{geom}}$ is the geometric area, while $A_{\mathrm{eff}} = G_{\mathrm{max}}\,\lambda ^2 / 4\pi$ is the effective area.

The main figures of merit are summarized in Table 2. The gain is relatively stable across the band, with a slight decrease for increasing frequency. The maximum value of 13.7 dB is obtained at 2.40 GHz, at which the aperture efficiency also reaches its maximum of $60\%$. At 2.44 and 2.48 GHz the peak gain is 13.3 and 12.1 dB respectively, with corresponding aperture efficiencies of $53\%$ and $41\%$. Energetic (Ohmic) efficiency was estimated by comparing measured gain and directivity, resulting in losses less than 1 dB over the whole bandwidth.

**Table 2:** Summary of measurements: $G_{\mathrm{max}}$ is the maximum gain, $\varepsilon _{\mathrm{ap}}$ is the aperture efficiency, HPBW is the Half Power Beam Width, SLL is the Sidelobe Level and $|S_{11}|$ is the input reflection coefficient.

Freq (GHz)	$\varvec G_{\mathrm{\bf max}}$ (dBi)	$\varvec \varepsilon _{\mathrm{\bf ap}}$ (%)	HPBW (°)	SLL (dB)	$|\varvec S_{\bf 11}|$ (dB)
2.40	13.7	60	46	− 15	− 12.5
2.44	13.3	53	48	− 13	− 15.6
2.48	12.1	41	56	− 10	− 10.5

**Figure 6:**
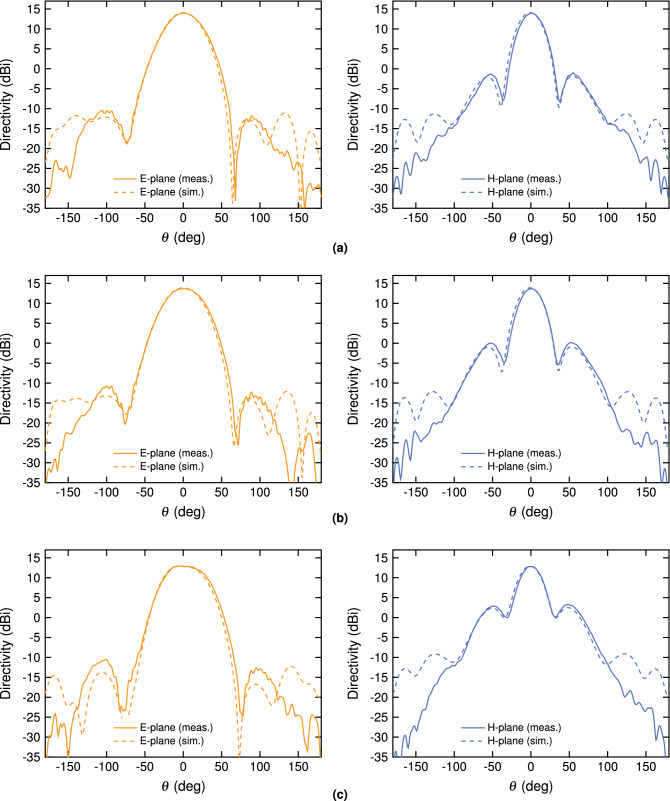
Simulated and measured directivity in the E- and H-plane for the optimized antenna at 2.40 GHz (**a**), 2.44 GHz (**b**) and 2.48 GHz (**c**).

Measured values of directivity in the E- and H-plane are shown in Fig. 6 for frequencies of 2.40, 2.44 and 2.48 GHz, corresponding to the lower, center and upper limit of the ISM band respectively. As is evident, the measurements compare very well with simulations, with negligible discrepancies $< 0.3$ dB in the main beam for all frequencies. The polarization of the radiated fields is purely linear on the symmetry plane, as enforced in the design, and therefore it is not shown here. In the entire main beam, the cross-polarization remains below $-20$ dB. Furthermore, design symmetry forces the radiation pattern in the H-plane to be symmetric. Sidelobe levels remain satisfactorily below $-25$ dB in the E-plane and $-13$ dB in the H-plane.

**Figure 7:**
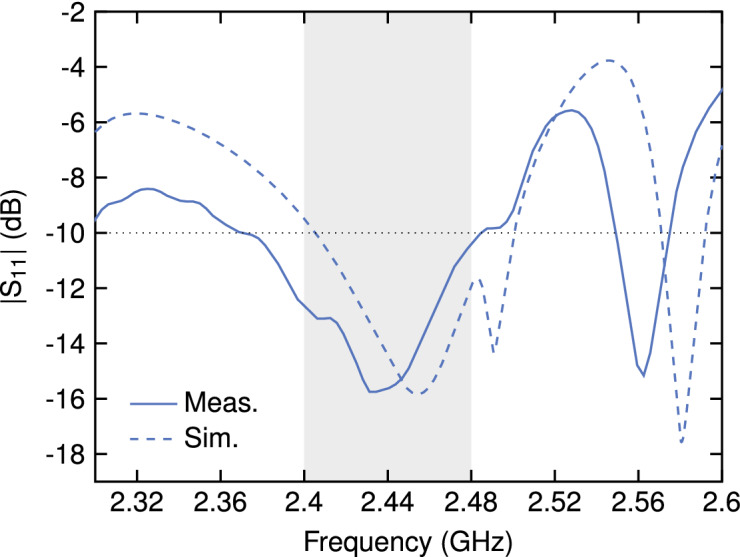
Measured and simulated input reflection coefficient magnitude.

Regarding the input reflection coefficient (Fig. 7), the proposed antenna shows a satisfactory matching over the whole design bandwidth, with values of $|S_{11}|$ below $-10$ dB and as low as $-15.6$ dB at the center frequency, where the optimization was performed. A comparison with simulated data showed a slight shift in the resonance peak, which can be attributed to the difficulties in modeling the feed accurately. Regardless of this, the reference bandwidth at $-10$ dB remains comparable in the two cases, with 86 MHz obtained from simulations and 109 MHz from measurements.

### Discussions

We have presented a novel procedure for the direct design of flat directive antennas. Surface shaping is obtained by inclusion/removal of hexagonal cells; this makes the optimization efficient, and free of manufacturing uncertainties. The design is based on a Genetic Algorithm combined with a boundary element electromagnetic solver.

The procedure was applied to the design of an antenna for the 2.4 GHz ISM frequency band. The antenna was fabricated and measured. Measurements confirmed the satisfactory results, notably an aperture efficiency as high as 60% and a gain higher than 13 dBi.

Because of the absence of a specific beam-forming network, we expect a favourable scaling of ohmic losses at higher frequencies, as in similar single-feed antennas. Therefore, it is interesting to put the presently measured aperture efficiency (on average, about 50%) in comparison with that of similar antennas based on metasurfaces. Results reported^28^ for a very high gain antenna in the X band (8.4 GHz) indicate efficiencies of the order of 35%, which is lower than the one achieved in our case, as expected at a higher frequency. Our significantly larger efficiency suggests a favourable projection for higher frequencies and larger gains.

The demonstrated robustness of the proposed method thus paves the way to machine design flat antennas with very high gain at high frequencies.

## Methods

### Design methodology

The electromagnetic simulation is based on our implementation of well known algorithms^14^ for the Method of Moments matrix computation, while the Genetic Algorithm has been implemented in MATLAB. The code has been deployed on a cluster with 24 computational nodes, and the optimization took 21 hours to reach convergence.

The combined requirement for maximum radiation along broadside and linear polarization corresponds to a Perfect Magnetic Conductor (PMC) boundary condition on a symmetry plane perpendicular to the surface. This was directly enforced in the build phase of the system matrix. No additional care was needed for the discretization, since the basis functions already forced the current to be tangential to the symmetry plane, as required by the PMC boundary condition.

For the purpose of reducing the computational effort, the optimization was carried out at a single frequency; we chose the centerband frequency 2.44 GHz of the targeted ISM band. The relatively stable performance over the entire band justified this choice.

The dielectric material backing the patterned metal layer was conceived as a mere mechanical support, and to allow its manufacturing with PCB technology. The flip-back layout was thus adopted to minimize field penetration in the dielectric. In this way, its final impact on electromagnetic phenomena was negligible, allowing the use of an efficient in-house solver which did not consider this dielectric layer.

Because of the above, the performance of the actual antenna was verified by full simulation prior to fabrication. This was done with a commercially available EM solver (CST Microwave Studio^29^); the simulated model incorporated all the details of the final design, i.e., inhomogeneity due to the dielectric substrate, thickness of the metalization, and details of the coaxial feed. A shift in the resonance frequency was observed with respect to the simplified model used during the optimization. Field analysis revealed a standing-wave behavior in one direction, and a traveling wave behavior in the orthogonal direction; this was expected because of wave physics and the imposed symmetry. Therefore, the geometry was subsequently refined by shrinking it in the direction corresponding to standing-wave resonance. This allowed to tune the resonance frequency (dotted line in Fig. 7) while preserving the radiation properties. It is important to stress that this final tweak was solely due to the neglect of the supporting dielectric layer during the design process, in turn due to the readily available in-house software. Therefore, it is not a limitation of the proposed approach.

### Measurement methodology

The realized antenna was measured in the Spherical Near Field (SNF) range facility installed in the anechoic chamber of the Politecnico di Torino (Turin, Italy). The size of the chamber is $5\times 5\times 4\,{\mathrm {m}}^3$, it is fully lined with 1800 absorbers, and it can be used for frequencies between 700 MHz and 40 GHz. The system was realized by Orbit FR (date of installation: July 2009). It is equipped for the measurements of antennas up to a maximum dimension of about 1.5 m and a weight of 30 kg.

The RF system is based on an Agilent PNA E8363B^30^ coupled to a commercial measurement software (MiDAS)^31^; the latter performs a NF to FF transformation via spherical wave expansion^32^. The field radiated from the Antenna Under Test (AUT) was sampled in amplitude and phase at appropriate angular intervals (depending on the frequency and of the radius of the sphere which encloses all sources). The scan radius, i.e., the distance between the center of rotation of the AUT and the phase center of the probes, is of about 2.5 m.

## Data Availability

The data that support the findings of this study are available from the corresponding author on request.

## References

[1] Epstein A, Wong JPS, Eleftheriades GV (2016). Cavity-excited Huygens' metasurface antennas for near-unity aperture illumination efficiency from arbitrarily large apertures. Nat. Commun..

[2] Binion JD, Lier E, Hand TH, Jiang ZH, Werner DH (2019). A metamaterial-enabled design enhancing decades-old short backfire antenna technology for space applications. Nat. Commun..

[3] Sievenpiper D, Zhang L, Broas RFJ, Alexopolous NG, Yablonovitch E (1999). High-impedance electromagnetic surfaces with a forbidden frequency band. IEEE Trans. Microw. Theory Tech..

[4] Maci S, Minatti G, Casaletti M, Bosiljevac M (2011). Metasurfing: addressing waves on impenetrable metasurfaces. IEEE Antennas Wirel. Propag. Lett..

[5] Holloway CL, Love DC, Kuester EF, Gordon JA, Hill DA (2012). Use of generalized sheet transition conditions to model guided waves on metasurfaces/metafilms. IEEE Trans. Antennas Propag..

[6] Fong BH, Colburn JS, Ottusch JJ, Visher JL, Sievenpiper DF (2010). Scalar and tensor holographic artificial impedance surfaces. IEEE Trans. Antennas Propag..

[7] Minatti G, Caminita F, Casaletti M, Maci S (2011). Spiral leaky-wave antennas based on modulated surface impedance. IEEE Trans. Antennas Propag..

[8] Faenzi M (2019). Metasurface antennas: new models, applications and realizations. Sci. Rep..

[9] Araque Quijano JL, Vecchi G (2009). Optimization of an innovative type of compact frequency-reconfigurable antenna. IEEE Trans. Antennas Propag..

[10] Johnson JM, Rahmat-Samii Y (1999). Genetic algorithms and method of moments (GA/MOM) for the design of integrated antennas. IEEE Trans. Antennas Propag..

[11] Cormos D, Loison R, Gillard R (2004). A multistructure method of moments for EM optimization. Microw. Opt. Technol. Lett..

[12] Arnaud-Cormos D, Loison R, Gillard R (2007). Fast multistructure method of moments combined with a genetic algorithm (MSMoM/GA) for efficient optimization of printed antennas. IEEE Antennas Wirel. Propag. Lett..

[13] Villegas F, Cwik T, Rahmat-Samii Y, Manteghi M (2004). A parallel electromagnetic genetic-algorithm optimization (EGO) application for patch antenna design. IEEE Trans. Antennas Propag..

[14] Gibson WC (2008). The Method of Moments in Electromagnetics.

[15] Delabie C, Villegas M, Picon O (1997). Creation of new shapes for resonant microstrip structures by means of genetic algorithms. Electron. Lett..

[16] Alatan L, Aksun M, Leblebicioglu K, Birand M (1999). Use of computationally efficient method of moments in the optimization of printed antennas. IEEE Trans. Antennas Propag..

[17] Choo H, Hutani A, Trintinalia L, Ling H (2000). Shape optimisation of broadband microstrip antennas using genetic algorithm. Electron. Lett..

[18] Chakravarty S, Mittra R, Williams N (2001). On the application of the microgenetic algorithm to the design of broad-band microwave absorbers comprising frequency-selective surfaces embedded in multilayered dielectric media. IEEE Trans. Microw. Theory Tech..

[19] Jafar-Zanjani S, Inampudi S, Mosallaei H (2018). Adaptive genetic algorithm for optical metasurfaces design. Sci. Rep..

[20] Cormos D, Loison R, Gillard R (2005). Fast optimization and sensitivity analysis of nonintuitive planar structures. IEEE Trans. Microw. Theory Tech..

[21] Zucchi, M., Giordanengo, G., Righero, M., Araque Quijano, J. L. & Vecchi, G. Optimization of a flat directive antenna for 2.4 GHz band using genetic algorithm. In *13th European Conference on Antennas and Propagation (EuCAP)* (2019).

[22] Johnson J, Rahmat-Samii V (1997). Genetic algorithms in engineering electromagnetics. IEEE Antennas Propag. Mag..

[23] Weile DS, Michielssen E (1997). Genetic algorithm optimization applied to electromagnetics: a review. IEEE Trans. Antennas Propag..

[24] Haupt RL, Werner DH (2007). Genetic Algorithms in Electromagnetics.

[25] IEEE Standard for Definitions of Terms for Antennas. *IEEE Std 145-2013 (Revision of IEEE Std 145-1993)*10.1109/IEEESTD.2014.6758443 (2014).

[26] Rao S, Wilton D, Glisson A (1982). Electromagnetic scattering by surfaces of arbitrary shape. IEEE Trans. Antennas Propag..

[27] https://www.isola-group.com/products/all-printed-circuit-materials/i-tera-mt40/.

[28] Faenzi M (2016). Realization and measurement of broadside beam modulated metasurface antennas. IEEE Antennas Wirel. Propag. Lett..

[29] https://www.3ds.com/products-services/simulia/products/cst-studio-suite/.

[30] https://www.keysight.com/en/pd-72321-pn-E8363B/pna-series.

[31] http://www.mvg-world.com/en/products/field_product_family/antenna-measurement-2/midas-measurement-software.

[32] Hansen JE (1988). Spherical Near-Field Antenna Measurements.

